# Effect of high-dose glucocorticoid treatment on human brown adipose tissue activity: a randomised, double-blinded, placebo-controlled cross-over trial in healthy men

**DOI:** 10.1016/j.ebiom.2023.104771

**Published:** 2023-09-04

**Authors:** Claudia Irene Maushart, Wenfei Sun, Alaa Othman, Adhideb Ghosh, Jaël Rut Senn, Jonas Gabriel William Fischer, Philipp Madoerin, Rahel Catherina Loeliger, Robyn Melanie Benz, Martin Takes, Christoph Johannes Zech, Alin Chirindel, Felix Beuschlein, Martin Reincke, Damian Wild, Oliver Bieri, Nicola Zamboni, Christian Wolfrum, Matthias Johannes Betz

**Affiliations:** a Department of Endocrinology, Diabetes and Metabolism, University Hospital Basel and University of Basel, Basel, Switzerland; b Institute of Food, Nutrition, and Health, ETH Zurich, Schwerzenbach, Switzerland; c Department of Biology, Institute of Molecular Systems Biology, ETH Zurich, Zurich, Switzerland; d Functional Genomics Center Zurich, ETH Zurich and University of Zurich, Zurich, Switzerland; e Department of Radiology and Nuclear Medicine, University Hospital Basel, University of Basel, Basel, Switzerland; f Department of Endocrinology, Diabetology and Clinical Nutrition, University Hospital Zurich (USZ) and University Zurich (UZH), Zurich, Switzerland; g Department of Medicine IV, University Hospital, LMU Munich, Munich, Germany

**Keywords:** Brown adipose tissue, Cold-induced thermogenesis, Glucocorticoids, Prednisone, Energy expenditure

## Abstract

**Background:**

Glucocorticoids (GCs) are widely applied anti-inflammatory drugs that are associated with adverse metabolic effects including insulin resistance and weight gain. Previous research indicates that GCs may negatively impact brown adipose tissue (BAT) activity in rodents and humans.

**Methods:**

We performed a randomised, double-blinded cross-over trial in 16 healthy men (clinicaltrials.govNCT03269747). Participants received 40 mg of prednisone per day for one week or placebo. After a washout period of four weeks, participants crossed-over to the other treatment arm. Primary endpoint was the increase in resting energy expenditure (EE) in response to a mild-cold stimulus (cold-induced thermogenesis, CIT). Secondary outcomes comprised mean ^18^F-FDG uptake into supraclavicular BAT (SUV_mean_) as determined by FDG-PET/CT, volume of the BAT depot as well as fat content determined by MRI. The plasma metabolome and the transcriptome of supraclavicular BAT and of skeletal muscle biopsies after each treatment period were analysed.

**Findings:**

Sixteen participants were recruited to the trial and completed it successfully per protocol. After prednisone treatment resting EE was higher both during warm and cold conditions. However, CIT was similar, 153 kcal/24 h (95% CI 40–266 kcal/24 h) after placebo and 186 kcal/24 h (95% CI 94–277 kcal/24 h, p = 0.38) after prednisone. SUV_mean_ of BAT after cold exposure was not significantly affected by prednisone (3.36 g/ml, 95% CI 2.69–4.02 g/ml, vs 3.07 g/ml, 95% CI 2.52–3.62 g/ml, p = 0.28). Results of plasma metabolomics and BAT transcriptomics corroborated these findings. RNA sequencing of muscle biopsies revealed higher expression of genes involved in calcium cycling. No serious adverse events were reported and adverse events were evenly distributed between the two treatments.

**Interpretation:**

Prednisone increased EE in healthy men possibly by altering skeletal muscle calcium cycling. Cold-induced BAT activity was not affected by GC treatment, which indicates that the unfavourable metabolic effects of GCs are independent from thermogenic adipocytes.

**Funding:**

Grants from Swiss National Science Foundation (PZ00P3_167823), Bangerter-Rhyner Foundation and from Nora van der Meeuwen-Häfliger Foundation to MJB. A fellowship-grant from the Swiss National Science Foundation (SNF211053) to WS. Grants from 10.13039/501100001659German Research Foundation (project number: 314061271-TRR 205) and Else Kröner-Fresenius (grant support 2012_A103 and 2015_A228) to MR.

**Research in context**

**Evidence before this study**

Based on *in vitro* experiments and preclinical studies in rodents, glucocorticoids (GC) have been claimed to inhibit cold-induced brown adipose tissue (BAT) thermogenesis. Two small trials which coincided with our trial investigated the effect of prednisone given at medium dosage (10–15 mg of prednisone) on BAT in humans. One trial showed higher BAT activity while the other trial revealed lower BAT activity. However, a substantial proportion of participants in the second trial had no relevant BAT activity at baseline.

**Added value of this study**

We performed a placebo controlled, double-blinded randomised, cross-over trial in 16 healthy male volunteers. We demonstrate that GC treatment increases resting energy expenditure (REE) but does not affect cold-induced BAT activity. Importantly, participants took a daily dose of 40 mg prednisone or placebo over a week which is considerably higher than in the previous trials but reflects a typical dosage used in clinical care. All participants were pre-screened to ensure that cold-induced BAT activity was present at baseline to ensure that we could find a potential reduction in BAT activity. Additionally, we used a stern cooling protocol to elicit maximum BAT activity while avoiding shivering. We corroborated our findings by analysing the plasma metabolome and gene expression in supraclavicular BAT. Moreover, we present data from an observational trial in patients taking GCs for at least six weeks which is in line with the findings from our interventional trial. Besides, we determined the expression of BAT markers in peri-adrenal adipose tissue of patients who underwent adrenalectomy for benign adrenal adenomas with and without autonomous cortisol secretion. Cortisol secretion did not affect gene expression in these patients.

**Implications of all the available evidence**

With our findings we can demonstrate that short term high-dose GC exposure does not affect BAT activity in humans. Nonetheless, REE was higher after GC exposure than after placebo.

## Introduction

Brown adipose tissue (BAT) is comprised of specialised cells that can generate heat in response to cold exposure or adrenergic stimuli.^1^^,^^2^ Its thermogenic activity is especially pronounced in small placental mammals and human infants. It helps to maintain core body temperature in a cool environment without the need for shivering. Beyond this important homeostatic function, which has been conserved during evolution, the presence of active BAT is associated with higher insulin sensitivity and lower levels of obesity.^3^ While previously thought to be existing exclusively in human infants, BAT has unambiguously been shown to be present in the majority of human adults.^4^^,^^5^ Importantly, thermogenic adipocytes can differentiate from previously white adipocytes in response to repetitive cold stimuli and thus allow the organism to adapt to lower environmental temperatures.^6^

Glucocorticoid (GC) dependent signalling plays a complex role in the differentiation of adipose tissues. The glucocorticoid receptor (GR) interacts with adipogenic transcription factors such as C/EBPβ or PPARγ2^7^ and accelerates adipogenesis. Excess levels of GCs have been shown to reduce thermogenic activity of brown adipocytes activity both *in vitro* and in animal models.^8^^,^^9^ As therapeutic agents, GCs are commonly used due to their high anti-inflammatory efficacy. Acutely, even high doses of GCs are usually well tolerated. On the other hand, medium or high amounts of GCs that are given on a chronic basis lead to unfavourable metabolic alterations. Typically, intra-abdominal fat mass increases, while skeletal muscle mass and insulin sensitivity are reduced.^10^^,^^11^ Studies in animals indicated that GCs can inhibit differentiation of white to brown adipocytes and reduce thermogenic function.^12^ So far, three clinical trials have investigated the effect of GCs on human BAT function, but with conflicting results.^13^, ^14^, ^15^

Here, we demonstrate that one week of high-dose GC treatment in healthy male volunteers does not change BAT activity despite increases in resting energy expenditure (REE). In line with these physiological measurements, we found no relevant changes in the transcriptome of human BAT. However, we could observe a higher expression of genes implicated in oxidative phosphorylation, calcium cycling and beta-oxidation in skeletal muscle.

## Material and methods

### Study protocol and participants

The study protocol of the interventional trial in healthy volunteers was approved by the regional ethics committee (Ethik-Kommission Nordwest-und Zentralschweiz) at the University of Basel (EKNZ 2016-01859) and registered at clinicaltrials.gov (NCT03269747) on September 1, 2017. We submitted two major amendments to the ethics committee. In August 2017, we submitted the first amendment for additional biopsies of the BAT and the SKM. Originally, we had planned a PET-CT in only half of the participants. Due to an additional source of funding, we were able to amend the plan for a PET-CT for all participants in July 2018. The study was conducted according to the Declaration of Helsinki and ICH-GCP. Participants provided written informed consent before taking part in any study related procedures. Sixteen healthy male volunteers were recruited and the study was performed in Basel, Switzerland, between September 2017 and April 2019. All participants that were enrolled into the study finished the study, there were no drop outs. The study was independently monitored for safety and accuracy by the clinical trials unit at the University Hospital Basel.

**Fig. 1:**
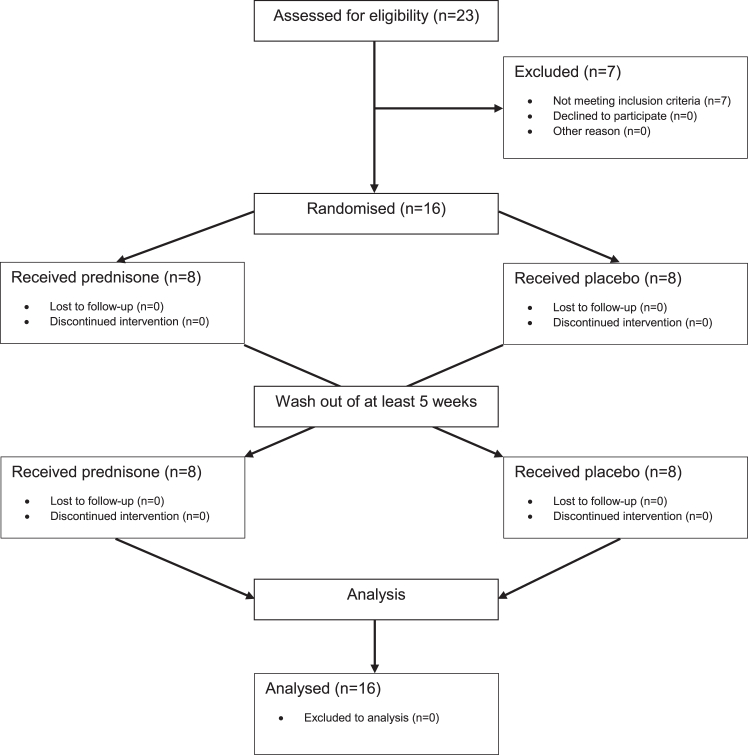
**CONSORT study diagram.** CONSORT study diagram describing the flow of participants in the randomized, cross-over trial.

Participants underwent a screening visit to rule out contraindications for study participation and to measure cold-induced thermogenesis (CIT). Only participants with a CIT above 5% of REE were enrolled into the study. They received 40 mg of prednisone over a period of seven days or matching placebo. The primary and secondary endpoints of the study were assessed on day 7 of the treatment. After a washout period of at least four weeks, participants crossed-over to the other treatment arm (Fig. 1). The sequence of prednisone or placebo treatment was randomised using a randomised block allocation (block size 4). Study medication was provided by the University Hospital’s pharmacy in sequentially numbered containers and both participants and investigators were blinded to the sequence until the study was completed.

In addition, we performed an observational study in patients treated with GCs. We measured energy expenditure (EE) and cold-induced BAT activity in patients treated with GCs for at least four weeks. Measurements were performed during the treatment phase and after patients were weaned from GCs and without GC for at least three months. The study protocol was approved by the institutional review board at the University of Basel (approval number EKNZ 2017-01742) and registered at clinicaltrials.gov (NCT03949361). The study was conducted according to the Declaration of Helsinki and ICH-GCP. Patients provided written informed consent before taking part in any study related procedures. They were recruited between June 2019 and March 2020. The study recruitment was closed early because of the COVID-19 pandemic.

### Indirect calorimetry

The study visits started at 8.30 am and all participants were fasted for at least 8 h prior to the study visit. EE was measured during 30 min of the warm phase and during the final 30 min of the cold phase using a ventilated-hood indirect calorimeter (Quark RMR, Cosmed, Rome Italy). The calorimeter was calibrated against a reference gas mixture (16% O_2_, 5% CO_2_, balance nitrogen) prior to each study visit.

### Mild cold exposure

In order to elicit CIT, participants underwent a controlled mild cold exposure. They were comfortably placed on a hospital bed wearing shorts and T-shirts and initially covered with a fleece blanket for measurements during warm conditions. The study room was air-conditioned with a stable ambient temperature of 24 °C year round. For cold exposure, the fleece blanket was removed and water perfused cooling mats were placed around the subject’s waist. Initially, the water temperature was set to 25 °C and was subsequently reduced at a rate of 1 °C every 2 min until a temperature of 10 °C was reached using a temperature controlled cooling device (Hilotherm clinic, Hilotherm GmbH, Argenbühl, Germany). The cold exposure was continued for a total of 120 min. Participants were closely observed for signs of shivering. In case of shivering, the water temperature was raised by 2 °C and the participants covered with a fleece blanket until shivering stopped.

### ^18^F-FDG-PET/CT imaging

After 120 min of cold exposure participants received a bolus of 75 MBq of ^18^F-Fluorodeoxyglucose (FDG). Following 30 min of uptake time, PET/CT acquisition was carried out on a Biograph mCT PET/CT scanner (Healthineers, Erlangen, Germany), as previously described.^16^ In short, PET/CT images from infero-orbital to tracheal bifurcation were acquired over 20 min in 2 bed positions. PET images were reconstructed using 3D ordered subset expectation maximization (OSEM) with 2 iterations and 28 subsets, using a post-reconstruction filter using an in-plane Gaussian convolution kernel with a FWHM of 5.0 mm. Low-dose CT (120 kV and 25 mA with CarDose4D) was used for anatomic landmark recognition and attenuation correction.

### Magnetic resonance imaging

One day after ^18^F-FDG-PET/CT, we performed magnetic resonance imaging (MRI) on a Magnetom Prisma 3 T scanner (Siemens Healthineers, Erlangen, Germany). Image acquisition lasted 6:26 min. Study participants wore shorts and t-shirt and were covered with a blanket. Images were acquired of the neck/shoulder area using a 3D volumetric, interpolated multi-echo spoiled gradient-echo (VIBE) sequence with monopolar readout, a repetition time of 13.50 ms, flip angle of 6°, 6-echos [1.09 ms, 3.19 ms, 5.29 ms, 7.39 ms, 9.49 ms, 11.59 ms] and a bandwidth of 810 Hz/Px for each. The image resolution parameters were as follows: Field of view: 256 mm (read) x 384 mm (phase); imaging matrix: 192 × 288 × 72; voxel size (mm): 1.3 × 1.3 x 1.3. Fat fraction was calculated with the vendors built in evaluation software using the mDixon-separation method.

### Tissue biopsies

In fifteen of the sixteen participants, biopsies were sampled from the supraclavicular adipose tissue depot at the site of most prominent FDG uptake and the *M. vastus lateralis*. An experienced interventional radiologist performed an ultrasound guided tru cut biopsy using a 17G coaxial needle and a 18G core biopsy needle and instrument (Bard, Magnum, Covington, G A) and took two core cylinders. The samples were immediately frozen and stored at −80 °C. For safety reasons, the study participant was monitored clinically for an additional 30 min after the intervention.

### Retroperitoneal adipose tissue samples

Retroperitoneal adipose tissue samples from the tumour bank at the Department of Internal Medicine IV at LMU Munich were analysed. Samples from 30 patients who had surgery for benign adrenal tumours were obtained. After removal of the tumour, the samples were put on ice and adjacent fat was removed and frozen in liquid nitrogen. The Ethical Review Board of the Ludwig-Maximilian-University (Munich, Germany, approval number: 379-10) approved of the study, which was part of the European Network for the Study of Adrenal Tumours registry and biobank initiative (www.ensat.org). All patients provided written informed consent.

### RNA extraction and library preparation

Gene expression analysis was performed in an exploratory manner without pre-defining certain genes of special interest. RNA was extracted from the scBAT and SKM biopsy samples from the interventional trial and the retroperitoneal adipose tissue samples from the adrenal tumour cohort.

RNA isolation and library preparation from tissue samples were performed as previously described.^17^ In brief, total RNA was extracted from frozen muscle and brown adipose tissue samples with TRI Reagent (15596018, Invitrogen), according to the manufacturer’s instructions. DNAse treatment was applied to eliminate genomic DNA. Quality of the RNA was determined using a TapeStation instrument (Agilent). All samples had an RNA integrity number (RIN) > 8. The rRNA was depleted, and purified RNA was used for the preparation of libraries using the TruSeq Stranded Total RNA (20020596, Illumina). Finally, libraries were sequenced on a Novaseq 6000 instrument (Illumina).

### RNA sequencing and gene expression analysis

RNA sequencing was performed at the Functional Genomics Center Zurich (FGCZ) on an Illumina NovaSeq instrument. Raw sequencing reads of all samples were processed and analysed using the SUSHI framework^18^^,^^19^ developed by FGCZ. Adapter sequences and low-quality bases were trimmed off from the raw reads using fastp v0.20^20^ for quality control. Filtered high quality reads were mapped against the human reference genome assembly (build GRCh38. p13) using STAR v2.7.4a. Gene expression values were quantified using the R package Rsubread v2.2.4^21^ and genes were considered to be present if they had at least 10 counts in half of the samples. Differential gene expression analysis was performed using the R package edgeR v3.34. For GlucoBAT cohort, the individual effect was included as a covariate into the negative binomial model of edgeR.^22^ Genes showing at least one-fold significant (p-value < 0.01; |log2FC| > 0.5) expression difference were considered to be differentially expressed. In addition, the false discovery rate (FDR) was calculated by applying the Benjamini-Hochberg method on the p-values. Gene set enrichment analysis was performed using the R package clusterProfiler v4.0.^23^ Normalised gene expression values in RPKM (Reads Per Kilobase of transcript per Million mapped reads) unit were used to perform the BATLAS^24^ deconvolution of adipocyte fractions.

### Plasma metabolome analysis

Before and after completion of cold exposure blood was sampled into serum gel tubes. After 30 min samples were centrifuged at 4 °C. Serum was separated and frozen at −80 °C until analysis.

Metabolite extraction was performed on the Hamilton STAR M liquid handling robot with the following protocol. In brief, 10 μl of the sample was aliquoted into 1.2 ml 96-deep well plates. Afterward, 300 ul of cold extraction solvent Methanol: H2O, (4:1 v:v) was added. After brief vortexing on a plate shaker, the samples were kept at −20 °C for 2 h. The samples were then centrifuged at 4000 g for 20 min. After centrifugation, the supernatant was transferred to another deep-well plate, and dried under N_2_. Before mass spectrometric analysis, the dried extracts were then resuspended in 100 ul MilliQ H2O and then transferred to PCR 96-well plates, and then sealed using heat sealing.

Flow injection mass spectrometry was performed as described previously.^25^ In brief, the samples were injected directly into the mass spectrometer (Agilent QTOF 6546) using an autosampler (Gerstel, USA) and a quaternary pump Agilent 1100 (Agilent, Germany). The samples were injected at an isocratic flow rate of 150ul/min of solvent isopropanol:H2O (6:4, v:v) that contains ammonium fluoride (1 mM) and the reference compounds hexakis (2,2,3,3-tetrafluoropropoxy) phosphazene) and Homotaurine (3-Amino-1-propane sulfonic acid). Electrospray ionization was used with the flowing source parameters: gas temperature 225 °C, drying gas 5 l/min, nebulizer 20 psi, sheath Gas Temperature 350 °C, sheath gas flow 10 l/min, Vcap 3500 V and Nozzle voltage 1000 V and Fragmentor was set 120 V, Skimmer to 65 V and the Oct1RF Vpp was set to 750 V. The mass spec was operated in negative full scan mode scanning the mass range (50–1000 m/z) at 1.4 spectra per second. Online mass correction using the reference masses 138.0230374 and 940.0003763.

Raw mass spectrometric data was converted to an open source format (mz5) and then processed using an in-house data processing pipeline (FiaMiner) in Matlab. The m/z axis for the whole dataset was recalibrated using expected masses. Afterwards, annotation was performed based on m/z, with mass accuracy of 0.001 Da matching to HMDB. Differential analysis statistics were performed in Matlab for pairwise comparisons followed by correction of multiple comparisons. Pathways enrichment analysis was performed in Matlab using the HMDB pathway definition v3.

### Statistics

#### Randomisation and blinding

Randomisation of the study sequence was performed by the Pharmacy of the University Hospital Basel using a randomised block procedure (block size 4). Study medication was provided in sealed, numbered containers. Containers and tablets were identical in appearance for prednisone and placebo. All investigators and participants were blinded to the allocation sequence until completion of the study and collection of the outcome data.

#### Sample size calculation

We used G∗Power (Version 3.1.9, University of Düsseldorf, Germany) to calculate the sample size.^26^ Based on *in vitro* studies and in vivo studies in mice we hypothesized that prednisone would reduce BAT thermogenesis by at least 33%. Based on prior studies^27^ we expected a correlation of 0.7 between the two measurements in each subject. We assumed a mean CIT of 210 kcal/24 h with an SD of 85 kcal/24 h for the placebo condition and a mean CIT of 140 ± 85 kcal/24 h for the glucocorticoid condition. Using these assumptions, we calculated a minimal sample size of 10 participants necessary to detect a statistically significant difference (standardized effect size 1.06, α = 0.05, 1-β = 0.80, two-sided testing with Wilcoxon signed-rank test). In order to account for heterogeneity of the sample and dropouts we decided to include 16 participants into the study. This allowed us to detect a clinically meaningful difference in BAT function in response to cold exposure with a power of >85%.

#### Study endpoints and analysis

Study related data was captured electronically (eCRF) using the secuTrial data capture system maintained by the clinical trials unit of the University Hospital Basel. The primary endpoint of the study was CIT after one week of prednisone as compared to placebo. Secondary endpoints were fat content of supraclavicular BAT (scBAT, determined by MRI), volume of scBAT (determined by MRI), supraclavicular skin temperature, scBAT activity determined by FDG-PET/CT (SUV_mean_). Two-way ANOVA was used to analyse the effect of prednisone and cold exposure on EE, followed by Holm-Šídák test for multiple comparisons. Pairwise comparisons of primary, secondary and explorative study outcomes were analysed by Wilcoxon-signed-rank tests or paired t-tests for non-normally distributed and normally distributed data, respectively. All tests were two-sided and a p-value below 0.05 was considered statistically significant. No corrections for multiple testing were applied. For the statistical analysis, the full analysis set (FAS) comprised all study participants who were randomly assigned to a treatment sequence. The complete analysis set comprised all participants who completed the study per protocol.

Wilcoxon signed-rank tests, t-tests and two-way ANOVAs were calculated and figures were created in GraphPad Prism Version 9 (GraphPad Inc., La Jolla, CA).

In order to test for the effect of co-variables and random effects on CIT and glucose uptake into BAT we used mixed-effects models in the statistical computing package R (Version 4.1^Ref.^^28^) and the package “nlme”.^29^ For the respective model CIT and SUVmean were the dependent variables. As continuous predictors, EE during warm temperature and the average outside temperature during the week before the study visit were included and the allocation to the study drug or placebo was included as categorical predictor (fixed effects). We expected considerable interindividual variability of both CIT and SUVmean and therefore added a random effect term, which assumed random intercepts for each individual. We used a compound symmetry covariance structure and the model was fitted by a restricted maximum likelihood (REML)-based repeated measures approach.

#### Role of funders

The funders of the study had no influence on its planning, conduction and analysis of the data.

## Results

### Participants

Between February 2018 and February 2019, we screened 23 young, healthy, male volunteers and measured cold-induced thermogenesis (CIT). In 16 subjects the basal metabolic rate increased by at least 5% in response to cold exposure. Only those were enrolled into the clinical trial. Participants were randomly assigned to a treatment sequence (placebo first or prednisone first). The two sub-groups did not differ in terms of baseline characteristics (Table 1). All enrolled participants completed the trial according to the protocol and the data from all participants was included in the final analysis (Fig. 1).

**Table 1:** Clinical characteristics at baseline.

	Baseline all	Placebo first	Verum first
Number	16	8	8
Age (years)	24.45 (22.11–26.79)	22.91 (19.43–26.38)	26.00 (22.40–29.59)
Sex (% male)	100	100	100
Weight (kg)	73.52 (69.01–78.02)	71.64 (62.60–80.67)	75.4 (70.96–79.84)
Height (cm)	180.7 (178.2–183.2)	179.0 (174.0–184.0)	182.4 (180.5–184.3)
BMI (kg/m^2^)	22.5 (21.3–23.7)	22.34 (19.74–24.94)	22.66 (21.57–23.75)
EE_warm_ (kcal/24 h)	1841 (1750–1932)	1828 (1671–1984)	1855 (1718–1992)
EE_cold_ (kcal/24 h)	2108 (1999–2218)	2105 (1930–2280)	2112 (1931–2293)
CIT	266.9 (196.2–337.7)	277.0 (175.5–378.5)	256.9 (130.7–383.0)
TSH (mU/L)	2.29 (1.70–2.88)	2.61 (1.73–3.48)	1.98 (1.02–2.93)
free T4 (pmol/l)	17.40 (16.47–18.33)	17.28 (16.30–18.25)	17.53 (15.62–19.43)
Triglycerides (mmol/l)	0.87 (0.73–1.01)	0.86 (0.64–1.08)	0.87 (0.64–1.10)
HbA1c (%)	4.94 (4.76–5.12)	4.85 (4.49–5.21)	5.03 (4.87–5.18)

All results are given as mean and 95% CI. The participants were equally randomized into the allocated group, multiple t-test comparison did not reveal any differences.

### Primary and secondary endpoints

The primary endpoint, CIT, did not differ between placebo and prednisone, 153 kcal/24 h (95% CI 40–266 kcal/24 h) and 186 kcal/24 h (94–277 kcal/24 h), respectively, p = 0.38, Table 2).

**Table 2:** Study outcomes.

Primary outcome	Placebo	Prednisone	Treatment difference	p-value
Cold-induced thermogenesis (kcal/24 h)	153 (40–266)	186 (94–277)	32 (−43 to 108)	0.38
**Secondary outcomes**				
MRI determined fat fraction of scBAT (%)	64.8 (61.6–67.9)	63.7 (59.8–67.5)	−1.6 (−3.6 to 0.5)	0.13
MRI determined volume of scBAT (cm^3^)	83.5 (69.1–97.9)	94.5 (76.9–112.1)	6.1 (−6.7 to 19.0)	0.32
Supraclavicular skin temperature in response to mild cold stimulus (°C)	35.4 (35.2–35.6)	35.7 (35.5–35.9)	0.30 (0.13–0.46)	0.0016
Cold stimulated FGD uptake in scBAT, SUV_mean_ (g/ml)	3.36 (2.69–4.02)	3.07 (2.52–3.62)	−0.29 (−0.79 to 0.21)	0.28
SUV_max_ in the scBAT depot (g/ml)	12.13 (8.19–16.07)	10.99 (7.61–14.38)	−1.14 (−4.29 to 2.02)	0.52

Data presented as mean and 95% CI; treatment difference: prednisone - placebo.

For the pre-specified secondary outcomes only the supraclavicular skin temperature was significantly lower after placebo treatment, 35.4 °C (95% CI: 35.2–35.6 °C), as compared to prednisone, 35.7 °C (95% CI, 35.5–35.9 °C, p = 0.0016). The results for the primary and secondary endpoints are given in Table 2.

### Safety

The study medication was well tolerated and no severe adverse events occurred. A list of the adverse events occurring during the study is given in Table 3. Five of the seven events were not related to the study drug. Two adverse events were possibly related to the study drug but resolved without sequel and did not necessitate stopping the study medication.

**Table 3:** Safety.

n = 7	Placebo	Verum	Prior to start of study drug
AE	1 (vasovagal reaction); 1 (fracture Dig 5, left hand)	1 (pain after BAT biopsy)	1 (common cold); 1 (sprained ankle)
AESI	1 (headache)	1 (nausea)	
SAE	0	0	0

All adverse events resolved spontaneously without sequel.

AE = Adverse event; AESI = Adverse event of special interest: Headache, abdominal symptoms, sleep related problems, oedema, psychic alterations; SAE = Serious adverse events.

### Energy expenditure in warm and cold environment

**Fig. 2:**
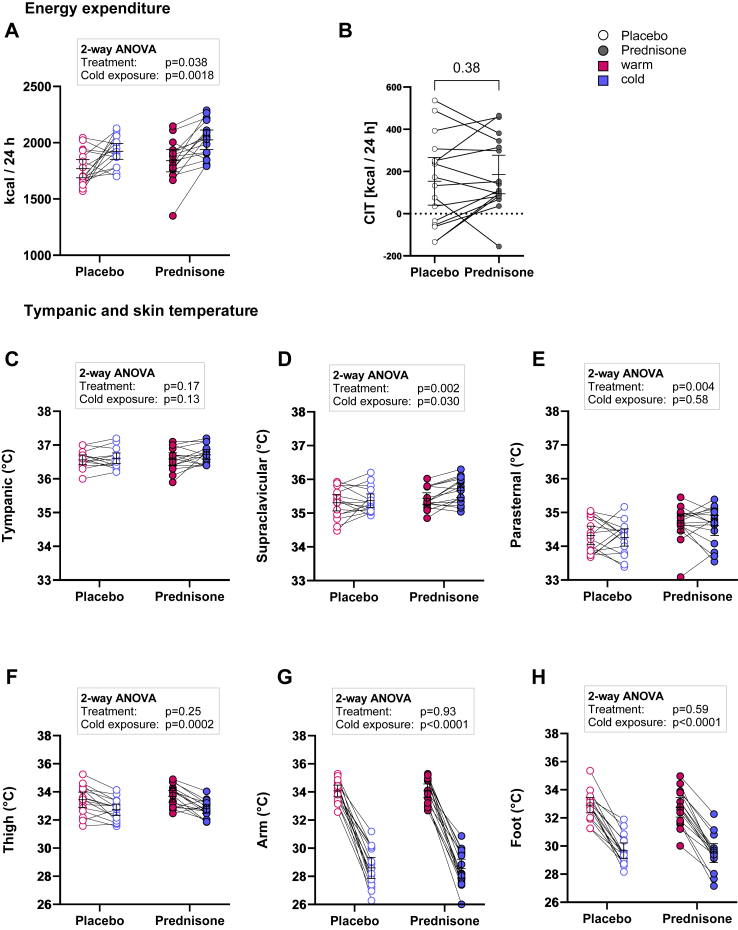
**Energy expenditure and temperature.** We measured energy expenditure (EE) during warm conditions (red) and after cold exposure (blue) once after intake of placebo (open circle) and once after prednisone treatment (filled circle). Cold exposure (p = 0.0018) and prednisone treatment (p = 0.038) significantly influenced EE (p for interaction = 0.38), data analysed by two-way ANOVA (n = 16) (A). However, cold-induced thermogenesis (CIT) defined as the change of EE after a cold stimulus was similar between the two groups (p = 0.38, paired t-test) (B). Tympanic temperature as a surrogate of core body temperature was not influenced by prednisone or cold exposure (C). Skin temperature in the supraclavicular region was significantly higher during prednisone as compared to placebo treatment and increased after cold exposure (D). Parasternal temperature was also higher during treatment with prednisone but not influenced by cold exposure (E). Peripheral skin temperature in the thigh, arm and foot region dropped significantly after cold exposure but not influenced by prednisone (F–H).

We measured EE both during ambient conditions and after 2 h of mild cold exposure. As expected, EE increased after cooling (two-way ANOVA, influence of cold exposure p = 0.0018). In comparison to placebo, a week of treatment with prednisone increased EE both at ambient temperature and after cold (influence of treatment p = 0.038, Fig. 2A). After placebo the mean EE_warm_ was 1769 kcal/24 h (95% CI: 1688–1851 kcal/24 h) and rose to 1923 kcal/24 h (95% CI: 1852–1993 kcal/24 h) after cold exposure. After the prednisone phase the mean EE_warm_ was 1840 kcal/24 h (95% CI: 1741–1940 kcal/24 h) and 2026 kcal/24 h (95% CI: 1939–2113 kcal/24 h) after cooling. In line with the findings for the primary endpoint, there was no interaction between prednisone treatment and cold exposure (p for interaction = 0.38). Cooling did not substantially affect glucose and insulin levels as well as thyroid hormones (Supplementary Table S1).

### Core body temperature and skin temperature

In parallel, we tested whether core body temperature was influenced by prednisone or mild cold exposure. Tympanic temperature as a surrogate marker of core body temperature was 36.6 °C (95% CI 36.4–36.7 °C) after placebo and 36.6 °C (95% CI 36.4–36.8 °C) after prednisone treatment. After cold exposure it remained at 36.6 °C (95% CI 36.5–36.8 °C) and 36.7 °C (95% CI 36.6–36.9 °C), respectively (2-way ANOVA p = 0.17 for treatment and p = 0.13 for cold exposure, Fig. 2C). Our findings are in line with previous data, which demonstrated that mild cold exposure does not reduce core body temperature but at the same time activates BAT thermogenesis.^1^ The main human BAT depot is located in the supraclavicular (SC) region.^30^ Supraclavicular (SC) skin temperature has been proposed as an indicator of BAT activity.^31^ SC temperature was 35.3 °C (95% CI 35.1–35.6 °C) after placebo during warm conditions and did not change after cooling (35.4 °C (95% CI 35.2–35.6 °C). After prednisone warm SC temperature was 35.4 °C (95% CI 35.3–35.6 °C) and rose to 35.7 °C (95% CI 35.5–35.9 °C, p = 0.002 for treatment and p = 0.030 for cold exposure; p = 0.14 for interaction, two-way ANOVA, Fig. 2D). As a reference for central skin temperature, we placed sensors in the parasternal region. In line with a higher REE after prednisone, the parasternal temperature during warm conditions was 34.3 °C (95% CI 34.0–34.6 °C) after placebo and 34.7 °C (95% CI 34.4–35.0 °C) after prednisone. It did not increase after cold exposure, 34.3 °C (95% CI 34.0–34.5 °C) and 34.6 °C (95% CI 34.3–34.9 °C), respectively (p = 0.004 for treatment, p = 0.58 for cold exposure, two-way ANOVA, Fig. 2E). The iButtons placed in peripheral skin locations (thigh, lower arm and foot) registered a substantial drop in temperature upon cold exposure, regardless of placebo or prednisone intake (p = 0.0002 and p < 0.0001 for cold exposure, Fig. 2F–H). Overall, these data indicate that the mild cold exposure was sufficient to elicit physiological responses aimed to maintain core body temperature.

### Imaging and glucose uptake into supraclavicular brown adipose tissue

**Fig. 3:**
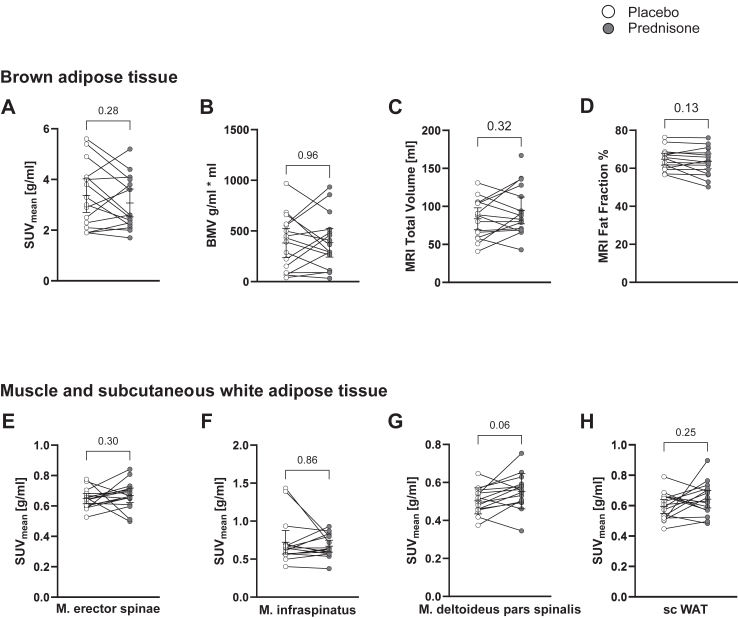
**Functional imaging of BAT, WAT and skeletal muscle.** Functional imaging of the supraclavicular region showed no significant change between the placebo and the prednisone treatment. In both settings the ^18^F-FDG-PET-CT of brown adipose tissue (BAT), which was performed after 2 h of cold exposure, showed comparable results for SUV_mean_ (p = 0.28, Wilcoxon signed rank test, n = 16) and for BAT metabolic volume (BMV, p = 0.96, paired t-test, A and B). Likewise, in the MRI scan total BAT volume and the fat fraction did not differ between the groups (C and D). The glucose uptake into skeletal muscle and white adipose in the neck region was unchanged after cold exposure (E–H).

BAT is a main driver but not the only determinant of CIT. In order to investigate directly the effect of GCs on BAT activity, we injected 75 MBq of ^18^F-fluorodeoxyglucose (FDG) intravenously after 120 min of cooling.^32^ We performed static PET-CT imaging 30 min after the injection and measured FDG activity as body weight corrected standardised uptake value (SUV) in the neck and upper thoracic region. In line with the calorimetry results, SUV_mean_ in scBAT as determined by BARCIST criteria^33^ did not differ between placebo and prednisone (3.36 g/ml (95% CI 2.69–4.02 g/ml) vs 3.07 g/ml (95% CI 2.52–3.62 g/ml), p = 0.28, Wilcoxon-signed rank test, Fig. 3A). SUV_max_ was 12.1 g/ml (95% CI 8.19–16.07 g/ml) and 11.0 g/ml (95% CI 7.61–14.38 g/ml; p = 0.52, Wilcoxon-signed rank test). Likewise, BAT metabolic volume was comparable between the two study conditions (382 g/ml∗ml, 95% CI 239–526 g/ml∗ml, vs. 385 g/ml∗ml, 95% CI 244–527 g/ml∗ml, p = 0.96, paired t-test, Fig. 3B). Active BAT primarily uses triglycerides to fuel heat production.^34^ Repetitive BAT activation leads to a lower fat content of the tissue.^35^ Accordingly, the fat fraction measured by 6-point DIXON MRI scanning in the scBAT depot has been described as a marker of BAT thermogenic capacity.^36^ We therefore assessed the scBAT depot by MRI scanning after each treatment period. The scBAT volume was 83.5 ml (95% CI 69.1–97.9 ml) after placebo and 94.5 ml (95% CI 76.9–112.1 ml) after prednisone (p = 0.32, paired t-test). The tissue’s fat fraction was similar after placebo (64.8%, 95% CI 61.6–67.9%) and after prednisone (63.7%, 95% CI 59.8–67.5%; p = 0.13, paired t-test, Fig. 3C and D).

### Glucose uptake into skeletal muscle and subcutaneous white adipose tissue

While BAT is metabolically highly active, it comprises a relatively small percentage of body volume. On the other hand, skeletal muscle (SKM) and subcutaneous white adipose tissue (scWAT) have a much larger volume and are contributing substantially to total EE. Therefore, we analysed whether the uptake of FDG into these tissues in the neck region is affected by prednisone treatment. In both, SKM and scWAT, prednisone treatment did not significantly change FDG uptake after cold exposure. SUV_mean_ in the M. erector spinae was 0.65 g/ml (95% CI 0.61–0.68 g/ml) after placebo and 0.67 g/ml (95% CI 0.62–0.72 g/ml) after prednisone (p = 0.30, Wilcoxon signed-rank test). SUV_mean_ in the M. infraspinatus was 0.72 g/ml (95% CI 0.57–0.88 g/ml) after placebo and 0.67 g/ml (95% CI 0.59–0.75 g/ml) after prednisone (p = 0.86, Wilcoxon signed-rank test). SUV_mean_ in the M. deltoideus was 0.50 g/ml (95% CI 0.47–0.54 g/ml) after placebo and 0.55 g/ml (95% CI 0.50–0.60 g/ml) after prednisone (p = 0.06, Wilcoxon signed-rank test). In the subcutaneous adipose tissue SUV_mean_ was 0.60 g/ml (95% CI 0.55–0.64 g/ml) after placebo vs. 0.64 g/ml (95% CI 0.59–0.70 g/ml) after prednisone, p = 0.25 (Wilcoxon signed-rank test), Fig. 3E–H.

### Mixed effects modelling of cold-induced thermogenesis and brown adipose tissue activity

BAT activity and CIT are influenced by numerous environmental factors and most importantly environmental temperatures.^37^^,^^38^ For this reason, we conducted the study visits exclusively from October to June, avoiding the summer months with higher average temperatures. In order to take into account the effects of outdoor temperature and other variables, we analysed the data using mixed-effects models. It is important to note that because we avoided the warm season in the study setup, CIT was not significantly influenced by outdoor temperature in our participants (p = 0.38). However, CIT was significantly correlated to the degree of REE during warm conditions (p < 0.0001). When REE was included into the statistical model, exposure to prednisone was significantly associated with higher CIT (p = 0.0004, see Supplementary Table S2). We then used the same statistical approach to investigate the effects of REE, outdoor temperature and prednisone treatment on SUV_mean_. Interestingly, BAT activity as determined by PET was independent of REE or prednisone treatment (Supplementary Table S3). These data underscore that the effects of prednisone treatment are independent of BAT activity.

### Gene expression in supraclavicular brown adipose tissue

**Fig. 4:**
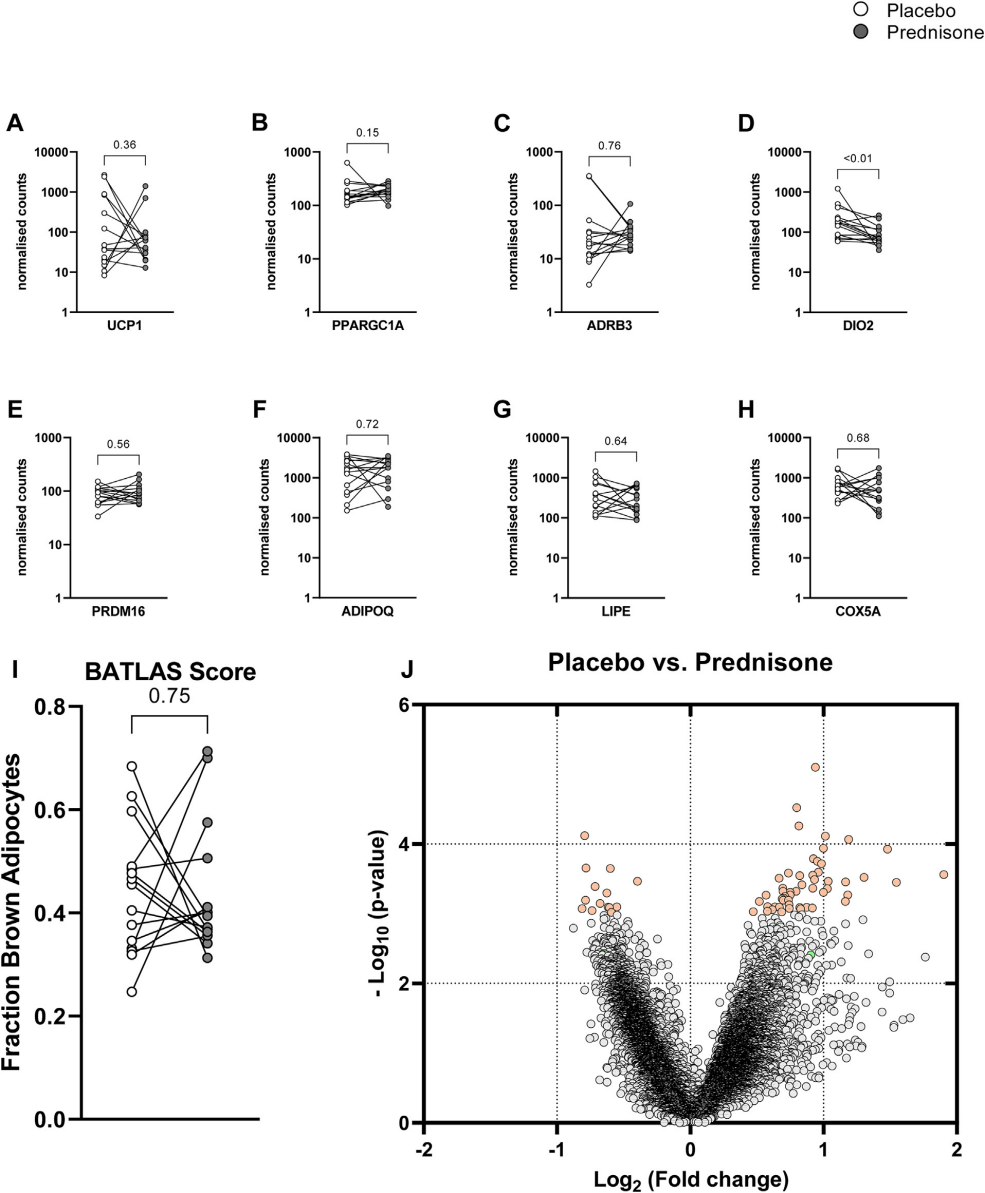
**Gene expression in supraclavicular BAT.** We analysed genes typically expressed in human BAT: Uncoupling protein 1 (UCP1), peroxisome proliferator activated receptor gamma co-activator 1α (PPARGC1A), β3-adrenergic receptor (ADRB3), deiodinase 2 (DIO2), PR domain containing 16 (PRDM16), the WAT marker adiponectin as well as hormone sensitive lipase (LIPE) and cytochrome c oxidase subunit 5a (COX5A) as a marker of mitochondrial content. With the exception of DIO2 (p < 0.01, Wilcoxon signed rank test, n = 15) normalized gene expression levels were comparable after placebo and prednisone (A–H). The BATLAS deconvolution score. Which can predict brown adipocyte content of a mixed tissue sample was practically equal after both treatments (0.44 ± 0.12 vs 0.44 ± 0.13, I), The volcano plot of the RNAseq data revealed that only few genes were differentially expressed at a significantly different level (orange hue, p < 0.001), However, the Benjamini-Hochberg corrected false-detection rate was above 0.1 for all analysed genes. DIO2 marked in light green.

In fifteen of the sixteen participants, we took biopsies of the scBAT depot after each treatment period and performed RNA sequencing. With the exception of DIO2 (p = 0.0043, paired t-test) classical markers of BAT were not significantly different between the prednisone and placebo periods (see Fig. 4A–H). Importantly, the fraction of brown adipocytes in the respective tissue samples as determined by BATLAS deconvolution score^24^ was not significantly different between placebo and prednisone exposure (Fig. 4I). The reduced level of DIO2 is most likely caused by lower plasma levels of TSH in response to GC exposure.^39^ A volcano plot of the expression data revealed that relatively few genes were expressed at a significance level of p < 0.001 or lower. However, the Benjamini-Hochberg corrected false-detection rate was above 0.1 for all analysed genes indicating that prednisone did not substantially affect scBAT transcription (Fig. 4J).

### Gene expression in skeletal muscle

**Fig. 5:**
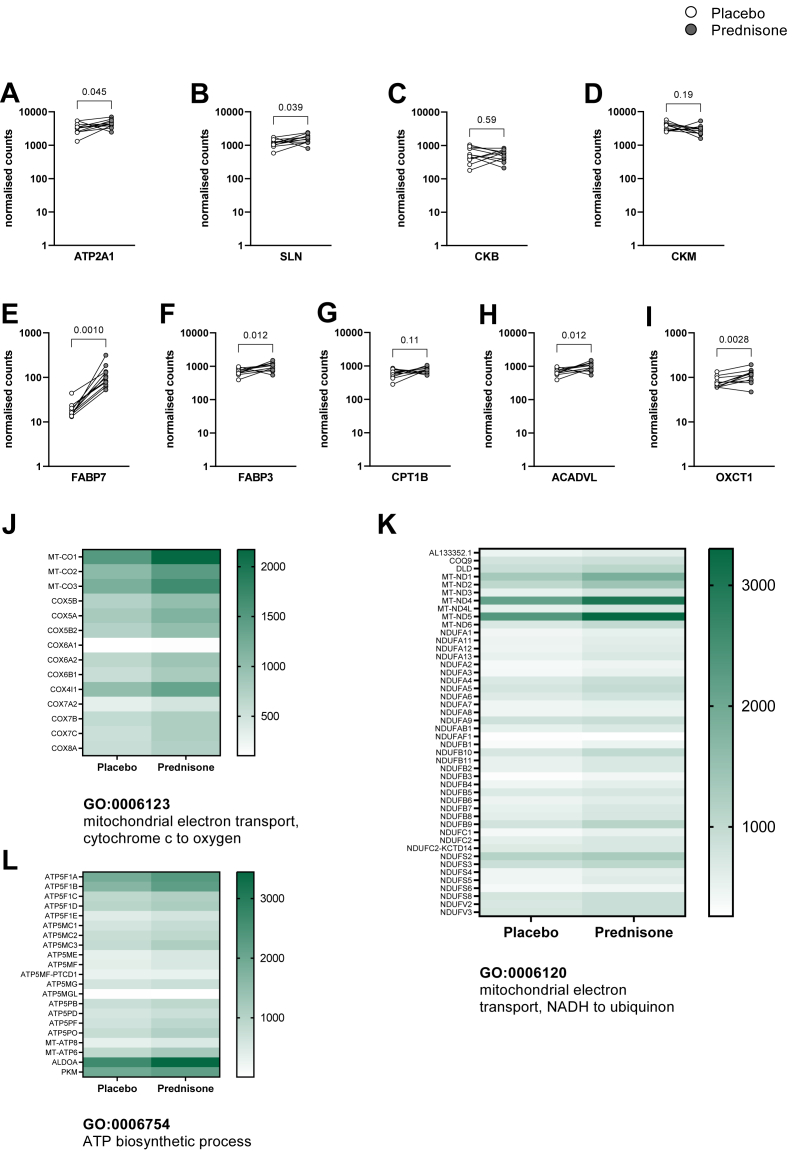
**Gene expression in skeletal muscle.** We analysed genes involved in calcium cycling and creatine metabolism. Expression of sarcoplasmic/endoplasmic reticulum calcium ATPase 1 (SERCA1, encoded by the ATP2A1) and sarcolipin (SLN) were slightly higher during treatment with prednisone (p = 0.045 and p = 0.039, respectively, both analysed with paired t-test, n = 11, A and B), suggesting increased calcium cycling. Creatine kinase B and M (CKB and CKM) were not differentially expressed between placebo and prednisone (p = 0.59, C, and p = 0.19, D, both paired t-test). Fatty acid binding proteins 7 and 3 (FABP7 and FABP3) were significantly increased after prednisone treatment (p = 0.001, E, Wilcoxon signed rank test and p = 0.012, F, paired t-test). Carnitine palmitoyltransferase 1 b (CPT1B), the rate-controlling enzyme of the long-chain fatty acid β-oxidation pathway in muscle mitochondria, showed an increased trend after prednisone treatment (p = 0.11, G, paired t-test). Mitochondrial very long-chain specific acyl-CoA dehydrogenase (ACADVL), which catalyses the first step of fatty acid oxidation, also higher expressed after prednisone (p = 0.012, H, paired t-test). 3-oxoacid CoA-transferase 1 (OXCT1), which catalyses the rate-limiting step in ketolysis expression was approximately 50% higher after prednisone (p = 0.0028, I, paired t-test). We performed gene set enrichment analysis (GSEA). Gene sets covering mitochondrial electron transport and ATP synthesis were among the top 15 gene sets in the GSEA suggesting increased mitochondrial respiratory activity (J–L).

In eleven participants we took core biopsies of the *M. vastus lateralis* and analysed gene expression (see Fig. 5). Besides UCP1 mediated uncoupling of oxidative phosphorylation several other futile cycles have been described during recent years. Calcium cycling in SKM has been described as a potential futile cycle which contributes to EE.^40^^,^^41^ Indeed, the expression level of SERCA1 (ATP2A1, p = 0.045) and sarcolipin (p = 0.039) were higher after treatment with prednisone than after placebo (Fig. 5A and B). Creatine cycling has been described as another important futile cycle, especially in beige adipose tissue.^42^^,^^43^ Levels of creatine kinase B and M were not significantly altered by prednisone (Fig. 5C and D).

GC treatment increased the expression of the fatty acid binding proteins FABP7 and FABP3 (p = 0.001 and p = 0.012; Fig. 5E and F) in SKM which suggested enhanced fatty acid transport to mitochondria. Indeed, carnitine palmitoyltransferase 1 b (CPT1b), the rate-controlling enzyme of the long-chain fatty acid β-oxidation pathway in muscle mitochondria, showed an upward trend after prednisone treatment (p = 0.11, Fig. 5G). Mitochondrial very long-chain specific acyl-CoA dehydrogenase (ACADVL), which catalyses the first step of fatty acid oxidation, was higher after prednisone (p = 0.012, Fig. 5H). Further, we found that 3-oxoacid CoA-transferase 1 (OXCT1), the rate-limiting enzyme of ketolysis, was significantly increased in SKM after prednisone as compared to placebo treatment (p = 0.0028, Fig. 5I, paired t-tests for all comparisons). Taken together, these findings speak for increased substrate flux from lipolysis into muscle mitochondria.

In order to elucidate potential effects of prednisone on muscle bioenergetics, we performed Gene Set Enrichment Analysis (GSEA, Supplementary Table S4). We found the top enriched gene sets to be associated with mitochondrial electron transport, as well as ATP synthesis. Intriguingly, these gene sets did not rank high in the GSEA of scBAT samples (Supplementary Table S5). The differences between the treatment periods were driven by a significantly higher expression of mitochondrial cytochrome c oxidase (MT-CO1, p = 0.026; MT-CO2, p = 0.023; MT-CO3, p = 0.022) as well as mitochondrial NADH-ubiquinone oxidoreductase (MT-ND1, p < 0.0001; MT-ND2, p = 0.012; MT-ND4, p < 0.0001; and MT-ND5, p < 0.0001) and ATP synthase F1 subunit α and β (ATP5F1 A, p = 0.0001; ATP5F1 B, p = 0.0013, paired t-tests).

### Plasma metabolome

**Fig. 6:**
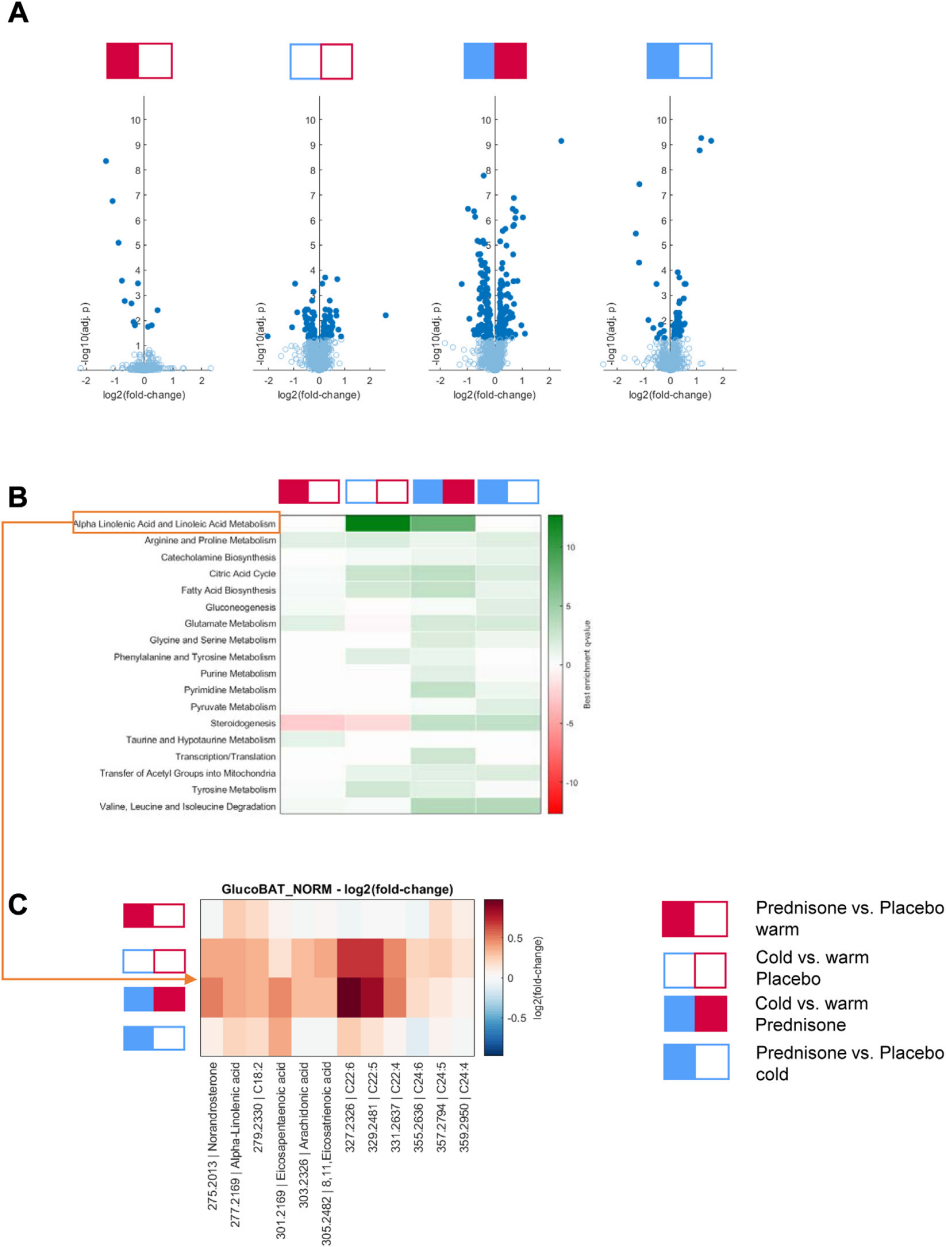
**Plasma metabolome.** Prednisone treatment altered levels of relatively few metabolites: Adrenal steroid hormones and their precursors were lower after prednisone (A, left panel). Cold exposure as compared to the warm condition had a pronounced effect on the metabolome both after placebo and prednisone treatment (A, central volcano blots). Pathway enrichment analysis showed significant changes in the metabolism of poly-unsaturated fatty acids (PUFA, B).

We sampled plasma before and after cold exposure at each study visit and performed untargeted plasma metabolomics. Prednisone treatment *per se* altered only a few metabolites. Adrenal steroid hormones and their precursors were lower after prednisone (Fig. 6A, left panel and Supplementary Fig. S1). In contrast, cold exposure had a more pronounced effect on the metabolome (Fig. 6A, central volcano plots). Short term cold stimulation has previously been shown to profoundly remodel lipid pathways in murine BAT.^44^ Pathway enrichment analysis revealed strong effects on the metabolism of poly-unsaturated fatty acids (PUFA) (Fig. 6B). Our results are in line with previous studies demonstrating an increase of PUFAs in response to BAT activation by cold.^45^, ^46^, ^47^ These findings indicate two important aspects of the experimental design: Study participants took the study medication as prescribed as it reduced endogenous steroidogenesis. Furthermore, cold induced changes to the metabolome were comparable after prednisone and placebo treatment. This corroborates our finding that GC treatment did not impede cold-induced BAT activity.

### Effect of long-term glucocorticoid treatment on brown adipose tissue activity

Additionally, we investigated a smaller cohort of seven patients who were treated with prednisone (average dosage >7.5 mg per day) for endocrine ophthalmopathy, inflammatory or rheumatoid disorders (EKNZ 2017-01742). We assessed CIT and BAT activity by ^18^F-FDG-PET-CT during treatment with prednisone and after prednisone was stopped for at least three months (n = 6 participants). In one patient, BAT activity was assessed when GC treatment was started and after three months. In line with the findings in healthy volunteers, values for CIT and FDG SUV_mean_ were comparable during the prednisone treatment and the control period. Of note, these individuals had much lower glucose uptake into BAT than the healthy volunteers (Supplementary Fig. S2).

### Effect of endogenous hypercortisolism on BAT markers in periadrenal fat

Aside from the supraclavicular depot, active BAT can be found in the retroperitoneal region.^48^^,^^49^ We therefore measured the expression of molecular markers for BAT activity and brown adipocyte differentiation in retroperitoneal adipose tissue samples from 30 patients. Tissue biopsies were obtained during surgery for benign adrenal tumours. We compared the results from eleven patients who had non-functioning adrenal adenomas or aldosterone producing adenomas (without cortisol co-secretion) to 19 patients who were suffering from hypercortisolism. With the exception of DIO2, markers of BAT differentiation and activity were similar in the group of hypercortisolemic vs. non-hypercortisolemic patients. The BATLAS score was comparable in both groups (Supplementary Fig. S3).

## Discussion

Based on preclinical studies using *in vitro* experiments and rodent models, glucocorticoids (GC) have been claimed to reduce brown adipose tissue (BAT) activity^8^^,^^50^ and increase its lipid content.^51^ In contrast, glucocorticoid receptor antagonists^52^ or inhibition of 11-β-hydroxysteroid dehydrogenase type I^53^ have been demonstrated to activate BAT and improve metabolic health in rodents. However, the relevance of these findings for human pathophysiology and metabolism are unclear.

Two trials in human subjects, which coincided with our study, investigated the effect of oral GCs on BAT activity in humans. The acute treatment over 24 h with a total dose of 30 mg prednisone led to higher cold-induced thermogenesis (CIT) and BAT activity as compared to placebo in a small cohort of six healthy men.^15^ A research team from Brisbane, Australia, treated eleven healthy volunteers with 15 mg of prednisone daily during a week. In their trial, ^18^F-FDG-uptake into BAT upon cold exposure was lower after the GC treatment as compared to placebo.^13^ Notwithstanding the different study designs, these two trials provided no clear indication on the effects of GCs on human BAT activity. Based on the pre-clinical findings in rodents and these two small trials, we expected that 40 mg of prednisone would reduce BAT activity.

In our current placebo controlled, randomised, cross-over trial we used a high daily dose of prednisone equivalent to ten times the physiologic production rate of cortisol per day. This treatment over the course of one week did not change the activity of human BAT. Several differences in experimental and cooling protocols between the three trials may explain the respective results.

The amount of prednisone used in our trial was almost three-fold higher than in the Australian study, which clearly impacts its metabolic effects.^13^ Based on the preclinical experiments we had hypothesised that GCs reduce BAT activity in a dose dependent fashion. In order to achieve a sufficiently pronounced treatment effect, we opted for 40 mg of prednisone which is considered a high dose in adults and commonly used to attenuate inflammation.^54^ Importantly, it clearly reflects the dose range commonly used in clinical settings. We observed a higher REE both during warm ambient temperature and after cold exposure. This is in line with previous data demonstrating that GC exposure acutely increases REE^55^^,^^56^ while cortisol withdrawal has the opposite effect.^57^ In the setting of mild cold exposure, a GC mediated increase in REE might provide sufficient heat production to maintain core temperature and thus reduce the need for additional BAT activity. Indeed, mixed-effects model analysis of our data revealed that a higher REE blunted CIT. This mechanism is in line with previous findings in patients suffering from hyperthyroidism.^58^

The normal room temperature in most animal facilities is around 21 °C and thus well below the thermoneutral zone of mice. Humans on the other hand live at thermoneutral temperatures most of the time. In a recent study, corticosterone reduced UCP1 protein levels in mice kept at 30 °C but not in those kept at 21 °C,^59^ This is in line with previous findings that cold exposure can ameliorate the detrimental effects of GCs on lipid metabolism.^50^ In human trials, the design of the cooling protocol substantially affects the amount of BAT activation.^60^ We exposed participants to water cooled blankets around the waist, which is a strong cold stimulus. This might be reflected by the resulting levels of BAT SUV_max_, which were two-fold higher in our cohort than in the Australian study that used cool room air to activate BAT.^13^ Additionally, lower environmental temperatures lead to a higher amount and activity of BAT and CIT in humans.^37^^,^^38^ It is of note, that the annual mean minimum temperature in Brisbane is 15.7 °C while the corresponding value for Basel, Switzerland, is 6.8 °C. In order to avoid seasonal blunting of BAT activity we performed all study visits between October and the beginning of June, avoiding the warm summer months. Therefore, we would like to speculate that the potential inhibitory effect of GCs on BAT might have been compensated by the cooler climate and sterner cooling protocol in our trial.

Currently, most clinical trials use ^18^F-FDG-PET/CT as a gold-standard to assess BAT activity. As recently demonstrated, FDG uptake into BAT can be blunted despite normal BAT activity.^61^ To overcome this limitation, we used a multimodal approach to determine the effect of GCs on BAT. CIT correlates well with BAT thermogenesis^62^^,^^63^ and did not differ significantly between the two treatments. The fat-fraction in scBAT is inversely correlated to BAT activity and can be determined by quantitative MRI (qMRI).^16^^,^^36^ In our trial, both the volume and the fat-fraction did not change. Additionally, we measured supraclavicular skin temperature as a marker of thermogenesis in the adjacent BAT depot.^31^^,^^64^ Cold exposure led to higher supraclavicular skin temperatures both, after placebo and GC treatment.

In addition to these physiological data, we analysed the molecular changes elicited by prednisone in scBAT with RNA sequencing. In line with the metabolic measurements, we did not observe statistically significant changes in BAT specific markers or the amount of brown adipocytes as determined by BATLAS score.^24^ The effects of GCs on adipocyte physiology have mainly been studied *in vitro* and in rodent experiments. Using a similar study design as ours, Ramshanker et al. investigated the effect of 37.5 mg of prednisone on lipolysis and insulin resistance in WAT of healthy human volunteers. Intriguingly, they observed a significant increase in the expression of CIDEA and ANGPTL4, which we did not see. Stimulation of WAT by prednisone increased lipolysis which contributed to elevated levels of plasma FFAs which might in turn fuel thermogenesis in BAT.^65^ Recent research indicates that cold exposure transiently reduces triglyceride levels while it robustly increases levels of FFAs especially levels of PUFAs.^66^ We also observed this cold-induced increase in PUFAs both after placebo and prednisone. As expected, steroid hormone levels were lower after prednisone than after placebo.

Data from experiments in mice indicate that continuous corticosterone exposure reduces BAT activity whereas an administration following the normal circadian rhythm does not alter CIT.^67^ In clinical practice and in our study, prednisone is given in the early morning in order to mimic the circadian rhythm of cortisol secretion. The outcome of our study might have been different, if the GC had been given in the evening or a GC with a longer half-life such as dexamethasone had been used. While dexamethasone is a selective glucocorticoid receptor (GR) agonist without activity at the mineralocorticoid receptor (MR), prednisone in a dosage of 40 mg per day activates both the GR and MR. Both pre-clinical studies^68^ and a clinical trial^69^ indicated that blocking the MR with spironolactone increased BAT activity. Given the design of our study we cannot dissect the GR-dependent effect of prednisone from the MR-dependent effects.

To support our findings from acute GC exposure of healthy volunteers we analysed two additional patient cohorts. We assessed BAT activity in patients who had been treated for at least three months with GCs and were then weaned from treatment. In this small cohort, BAT activity and CIT were not different during GC treatment as compared to the control period. BAT activity was generally lower than in healthy volunteers. Moreover, we analysed retroperitoneal adipose tissue samples from patients undergoing adrenal surgery. Patients suffering from endogenous GC excess did not exhibit different amounts of retroperitoneal BAT as compared to patients with other benign adrenal masses. These findings are in line with two prospective studies which investigated the metabolic effects of glucocorticoid treatment in patients suffering from rheumatoid arthritis^70^ and in patients presenting with Cushing’s syndrome.^71^

Prednisone treatment increased the REE which has been described previously.^55^^,^^56^ In order to further elucidate the effects of GC treatment on energy metabolism we performed RNA sequencing of *M. vastus lateralis* biopsies. In comparison to human BAT, skeletal muscle makes up a large proportion of total body weight. Calcium cycling via the Ca^2+^-ATPase SERCA1 and its regulator sarcolipin^72^ has been described as a futile cycle contributing to REE. Intriguingly, SERCA1 and sarcolipin mRNA levels were higher after prednisone treatment. This mechanism might contribute to the higher REE during prednisone treatment. Furthermore, we observed higher expression of genes involved in β-oxidation and mitochondrial electron transport after prednisone as compared to placebo.

We cannot exclude low grade shivering during the cooling as we did not use electromyography but observed and asked participants for signs and symptoms of cold-induced shivering. While EMG measurements can identify shivering earlier than visual assessment or measurement of EE, a direct comparison of these methods with EMG performed well to reliably detect shivering^73^ Furthermore, EMG has limited accuracy in monitoring deep muscle activity, especially in the neck region. This activity correlates with FDG-uptake into these muscle groups which was generally low in our study.^74^ One might consider that muscle shivering induced the transcriptional changes in muscle. Previously, the effect of cold-exposure on SKM in humans was investigated in healthy volunteers^6^ and patients with type 2 diabetes.^75^ In these studies, participants were repeatedly cold-exposed and shivering was induced. Importantly, cold-exposure did not alter SKM respiration and did not influence the amount of sarcolipin or SERCA2.^75^ Moreover, in both treatment arms participants were exposed to the same cold stimulus prior to the sampling of muscle tissue. The only experimental difference was the study drug intervention. The observed differences in gene expression are therefore most likely due to prednisone as compared to placebo. Taken together, these observations suggest that SKM reacts to GC exposure with increased futile cycling. However, further investigations on a functional level are needed to corroborate these findings.

Our study is limited by the fact that we only recruited male participants and we can therefore not draw any conclusions on the effect of GCs on BAT in women. The sample size was reasonable for a study investigating basic metabolic physiology but is of course much smaller than in an epidemiologic cohort study. Given the design of the trial, we can only speculate on the reasons behind the increase in REE and central skin temperature. Glucocorticoids increase simultaneously both lipolysis and triglyceride synthesis in white adipocytes.^76^ Futile lipid cycling has recently been demonstrated to contribute to heat production in BAT in the absence of UCP1.^77^ High dose prednisone drives proteolysis in human muscle^78^ and higher amino acid turnover was evident in the plasma metabolome after prednisone treatment in our study. The energetic cost of proteolysis contributes to total EE.^79^ Moreover, increased levels of glucagon^80^ could increase EE via hepatic mechanisms.^81^

Our trial has several strengths: Our cohort size was larger than that of previous studies and guided by sample size calculations. This gives the study sufficient power to detect clinically relevant changes in BAT activity. As we expected a reduction in BAT activity, we pre-screened participants for substantial levels of CIT, thereby ensuring at least moderate cold-induced BAT activity was present. In addition, we avoided the warm summer months to reduce confounding effects. In line with previous studies, we analysed FDG uptake into BAT. This parameter has recently been challenged as a surrogate marker of BAT activity as it may be altered despite normal BAT function.^61^ However, we assessed the effects of GCs with several complementing techniques and measured CIT, supraclavicular skin temperature as well as the volume of scBAT and its fat content by qMRI. In addition, we evaluated molecular changes by RNA sequencing and plasma metabolomics.

In conclusion, short-term treatment with prednisone increased EE in healthy men possibly by altering SKM calcium cycling. Cold-induced BAT activity was not affected by GC treatment which indicates that the unfavourable metabolic effects of GCs are independent from thermogenic adipocytes. Future work should investigate how GCs increase resting EE.

## Contributors

Conceptualisation, CIM, MJB; Study Design and Protocol: CIM, MJB; Data Collection/Experiments: CIM, WFS, JRS, PM, RCL, RMB, MT, CJZ, AC, FB; Data Analysis, CIM, AG, JGWF, PM, AO, OB; Formal Analysis, CIM, MJB; Writing CIM, MJB; Visualization, CIM, AO, MJB.; Supervision, MJB, CW, NZ, DW, OB, MR.

CIM and MJB have verified the underlying data. All authors commented on the manuscript and approved the final version of the manuscript.

## Data sharing statement

RNA Seq data were uploaded to GeneExpressionOmnibus (GEO) and can be accessed via: https://www.ncbi.nlm.nih.gov/geo/query/acc.cgi?acc=GSE220158 using the token “ozajcokcnbkbrqb”.

Individual participant data cannot be submitted to an open repository due to data protection laws. Metadata describing the type, size and content of the datasets will be shared along with the study protocol on the public repository dataverse.harvard.edu (https://doi.org/10.7910/DVN/1MTY6O).

Researchers can file a formal request to access the individual participant data to the Department of Clinical Research (DCR) at the University Hospital Basel. The DCR as independent data access committee will answer formal request of applicants, review and submit the project documents to the responsible ethics committee(s) and (upon approval) securely transfer the requested data to the applicants.

## Declaration of interests

The authors declare no conflicts of interest.
