# Supplementary material for: Effect of high-dose glucocorticoid treatment on human brown adipose tissue activity: a randomised, double-blinded, placebo-controlled cross-over trial in healthy men
Source: eBioMedicine. 2023 Sep 4;96:104771. doi: 10.1016/j.ebiom.2023.104771 (PMC10483510; doi:10.1016/j.ebiom.2023.104771)
Supplement: Supplementary Tables S1–S5 [file mmc1.docx]

**Supplementary table 1: Metabolic parameters**

| Parameter | Temperature | Placebo | Verum | Treatment difference | p-value |
| --- | --- | --- | --- | --- | --- |
| Glucose (mmol/l) | RT/warm | 4.88 (4.69 to 5.06) | 4.75 (4.35 to 5.15) | -0.13 (-0.51 to 0.26) | 0.50 |
|  | after cooling | 4.74 (4.50 to 4.98) | 5.06 (4.76 to 5.36) | 0.33 (0.0078 to 0.65) | 0.046 |
| Insulin (mIU/l) | RT/warm | 7.99 (6.78 to 9.20) | 10.18 (7.55 to 12.82) | 2.19 (-1.02 to 5.41) | 0.17 |
|  | after cooling | 5.52 (3.39 to 7.65) | 8.04 (5.49 to 10.60) | 2.52 (-0.41 to 5.45) | 0.083 |
| TSH (mIU/l) | RT/warm | 2.71 (2.00 to 3.42) | 2.45 (1.62 to 3.27) | -0.26 (-0.92 to 0.39) | 0.40 |
|  | after cooling | 1.68 (1.17 to 2.18) | 1.24 (0.81 to 1.67) | -0.44 (-0.89 to 0.018) | 0.058 |
| Free T4 (pmol/l) | RT/warm | 17.04 (16.26 to 17.82) | 17.17 (16.31 to 18.02) | 0.13 (-0.79 to 1.04) | 0.78 |
|  | after cooling | 17.44 (16.41 to 18.46) | 18.26 (17.26 to 19.26) | 0.82 (-0.45 to 2.09) | 0.18 |
| Free T3 (pmol/l) | RT/warm | 5.53 (5.18 to 5.89) | 5.24 (4.80 to 5.68) | -0.29 (-0.75 to 0.16) | 0.19 |
|  | after cooling | 5.66 (5.30 to 6.03) | 5.41 (4.87 to 5.95) | -0.25 (-0.84 to 0.33) | 0.36 |
| Triglycerides (mmol/l) | RT/warm | 1.40 (1.08 to 1.71) | 1.22 (0.93 to 1.50) | -0.18 (-0.49 to 0.13) | 0.20 |
| BMI (kg/m^2^) | RT/warm | 22.5 (21.2 to 23.8) | 22.5 (21.3 to 23.8) | 0.06 (-0.22 to 0.33) | 0.67 |

Mean (95% confidence interval)

RT= room temperature

**Supplementary table 2: Linear mixed effects model for relative CIT**

Random effects:

Formula: ~1 | Subject_ID

Residual

StdDev (Intercept): 0.672 (0.457 to 0.990)

Fixed effects: relCIT ~ as.factor(Prednisone) + Outdoor Temperature + Resting Energy expenditure

All continuous variables were z-scaled

|  | **Value** | **95% CI** | **DF** | **t-value** | **p-value** |
| --- | --- | --- | --- | --- | --- |
| **(Intercept)** | -0.163 | -0.522 to 0.196 | 45 | -0.92 | 0.36 |
| **Prednisone** | 0.326 | 0.153 to 0.499 | 45 | 3.79 | 0.0004 |
| **Outdoor Temperature (7d avg. max. temp.)** | -0.048 | -0.158 to 0.061 | 45 | -0.89 | 0.38 |
| **Resting Energy Expenditure** | -0.492 | -0.642 to -0.341 | 45 | -6.57 | <0.001 |

**Conditional R^2^: 0.87**

**Marginal R^2^: 0.31**

**Supplementary Table 3: Linear mixed effects model for SUV_mean_**

Random effects:

Formula: ~1 | Subject_ID

StdDev (Intercept) Residual 0.879 (0.592 to 1.31)

Fixed effects: SUVmean ~ as.factor (Prednisone) + Outdoor Temperature + Resting Energy Expenditure

|  | **Value** | **95% CI** | **DF** | **t-value** | **p-value** |
| --- | --- | --- | --- | --- | --- |
| **(Intercept)** | -0.015 | -0.494 to 0.465 | 45 | -0.062 | 0.95 |
| **Prednisone** | 0.029 | -0.239 to 0.298 | 45 | 0.22 | 0.84 |
| **Outdoor Temperature (7d avg. max. temp.)** | -0.0017 | -0.171 to 0.168 | 45 | -0.020 | 0.98 |
| **Resting Energy Expenditure** | -0.070 | -0.299 to 0.159 | 45 | -0.61 | 0.54 |

**Supplementary Table 4: Gene Set Enrichment Analysis of Skeletal Muscle Samples (sorted by q-value): GO Set Cellular compartment, Top 30 GO Terms. Placebo vs. Verum.**

| **ID** | **Description** | **setSize** | **enrichmentScore** | **NES** | **pvalue** | **p.adjust** | **qvalues** |
| --- | --- | --- | --- | --- | --- | --- | --- |
| GO:0005753 | mitochondrial proton-transporting ATP synthase complex | 18 | -0.956558231 | -2.68202413 | 1E-10 | 2.69E-09 | 1.6632E-09 |
| GO:0022625 | cytosolic large ribosomal subunit | 58 | -0.90934274 | -3.23917217 | 1E-10 | 2.69E-09 | 1.6632E-09 |
| GO:0015935 | small ribosomal subunit | 25 | -0.906154453 | -2.73794698 | 1E-10 | 2.69E-09 | 1.6632E-09 |
| GO:0022627 | cytosolic small ribosomal subunit | 44 | -0.902626488 | -3.0487978 | 1E-10 | 2.69E-09 | 1.6632E-09 |
| GO:0042788 | polysomal ribosome | 31 | -0.898797895 | -2.84699695 | 1E-10 | 2.69E-09 | 1.6632E-09 |
| GO:0070469 | respirasome | 64 | -0.893706682 | -3.21864507 | 1E-10 | 2.69E-09 | 1.6632E-09 |
| GO:0005747 | mitochondrial respiratory chain complex I | 52 | -0.855312702 | -3.0017823 | 1E-10 | 2.69E-09 | 1.6632E-09 |
| GO:0005840 | ribosome | 203 | -0.809764171 | -3.40191404 | 1E-10 | 2.69E-09 | 1.6632E-09 |
| GO:0000502 | proteasome complex | 63 | -0.769378652 | -2.77188068 | 1E-10 | 2.69E-09 | 1.6632E-09 |
| GO:0030017 | sarcomere | 61 | -0.752640919 | -2.70372004 | 1E-10 | 2.69E-09 | 1.6632E-09 |
| GO:0005762 | mitochondrial large ribosomal subunit | 56 | -0.745294476 | -2.63718048 | 1E-10 | 2.69E-09 | 1.6632E-09 |
| GO:0005743 | mitochondrial inner membrane | 431 | -0.702932121 | -3.16712949 | 1E-10 | 2.69E-09 | 1.6632E-09 |
| GO:1904813 | ficolin-1-rich granule lumen | 121 | -0.643514409 | -2.56022547 | 1E-10 | 2.69E-09 | 1.6632E-09 |
| GO:0034774 | secretory granule lumen | 106 | -0.637481671 | -2.49321666 | 1E-10 | 2.69E-09 | 1.6632E-09 |
| GO:0031966 | mitochondrial membrane | 151 | -0.604557828 | -2.48105981 | 1E-10 | 2.69E-09 | 1.6632E-09 |
| GO:1990904 | ribonucleoprotein complex | 156 | -0.579409851 | -2.38311577 | 1E-10 | 2.69E-09 | 1.6632E-09 |
| GO:0005681 | spliceosomal complex | 158 | -0.571231602 | -2.35213691 | 1E-10 | 2.69E-09 | 1.6632E-09 |
| GO:0005759 | mitochondrial matrix | 360 | -0.570031435 | -2.52944629 | 1E-10 | 2.69E-09 | 1.6632E-09 |
| GO:0005925 | focal adhesion | 399 | -0.500188169 | -2.23957012 | 1E-10 | 2.69E-09 | 1.6632E-09 |
| GO:0005882 | intermediate filament | 97 | 0.58854643 | 2.58239624 | 1E-10 | 2.69E-09 | 1.6632E-09 |
| GO:0030018 | Z disc | 126 | -0.570918522 | -2.2807751 | 1.5552E-10 | 3.9843E-09 | 2.4634E-09 |
| GO:0000276 | mitochondrial proton-transporting ATP synthase complex, coupling factor F(o) | 11 | -0.957057784 | -2.34302113 | 1.88E-10 | 4.5976E-09 | 2.8426E-09 |
| GO:0005763 | mitochondrial small ribosomal subunit | 29 | -0.813220311 | -2.54997538 | 2.0779E-10 | 4.8606E-09 | 3.0052E-09 |
| GO:0016529 | sarcoplasmic reticulum | 63 | -0.672301923 | -2.42213728 | 4.4516E-10 | 9.979E-09 | 6.1697E-09 |
| GO:0005758 | mitochondrial intermembrane space | 81 | -0.627563439 | -2.35843471 | 6.504E-10 | 1.3997E-08 | 8.6537E-09 |
| GO:0005741 | mitochondrial outer membrane | 170 | -0.51167432 | -2.11746079 | 6.7756E-10 | 1.402E-08 | 8.6684E-09 |
| GO:0031674 | I band | 23 | -0.841752367 | -2.5017167 | 1.0445E-09 | 2.0812E-08 | 1.2867E-08 |
| GO:0015934 | large ribosomal subunit | 19 | -0.864207372 | -2.43798964 | 2.7441E-09 | 5.2726E-08 | 3.2599E-08 |
| GO:0045095 | keratin filament | 45 | 0.669322041 | 2.55347131 | 4.3888E-09 | 8.1419E-08 | 5.0339E-08 |
| GO:0030016 | myofibril | 43 | -0.717834902 | -2.41544048 | 5.7402E-09 | 1.0294E-07 | 6.3645E-08 |

**Supplementary Table 5: Gene Set Enrichment Analysis of supraclavicular BAT samples (sorted by q-value): GO Set Cellular compartment, Top 30 GO Terms. Placebo vs. Verum.**

| **ID** | **Description** | **setSize** | **enrichmentScore** | **NES** | **pvalue** | **p.adjust** | **qvalues** |
| --- | --- | --- | --- | --- | --- | --- | --- |
| GO:0071013 | catalytic step 2 spliceosome | 82 | 0.592933572 | 2.55259148 | 1E-10 | 3.6533E-09 | 1.986E-09 |
| GO:1904813 | ficolin-1-rich granule lumen | 123 | 0.53368789 | 2.46690572 | 1E-10 | 3.6533E-09 | 1.986E-09 |
| GO:0005925 | focal adhesion | 399 | 0.410409089 | 2.17938875 | 1E-10 | 3.6533E-09 | 1.986E-09 |
| GO:0005765 | lysosomal membrane | 305 | 0.425176121 | 2.19183759 | 1E-10 | 3.6533E-09 | 1.986E-09 |
| GO:0005764 | lysosome | 388 | 0.417455815 | 2.20783272 | 1E-10 | 3.6533E-09 | 1.986E-09 |
| GO:0042470 | melanosome | 98 | 0.602449131 | 2.69688701 | 1E-10 | 3.6533E-09 | 1.986E-09 |
| GO:0005743 | mitochondrial inner membrane | 433 | 0.626980544 | 3.35902385 | 1E-10 | 3.6533E-09 | 1.986E-09 |
| GO:0005762 | mitochondrial large ribosomal subunit | 56 | 0.681235735 | 2.72324036 | 1E-10 | 3.6533E-09 | 1.986E-09 |
| GO:0005759 | mitochondrial matrix | 362 | 0.505423939 | 2.65549586 | 1E-10 | 3.6533E-09 | 1.986E-09 |
| GO:0005747 | mitochondrial respiratory chain complex I | 52 | 0.691774113 | 2.72661933 | 1E-10 | 3.6533E-09 | 1.986E-09 |
| GO:0016607 | nuclear speck | 390 | 0.427886053 | 2.25997313 | 1E-10 | 3.6533E-09 | 1.986E-09 |
| GO:0070469 | respirasome | 64 | 0.694225709 | 2.82902659 | 1E-10 | 3.6533E-09 | 1.986E-09 |
| GO:0005840 | ribosome | 203 | 0.575543058 | 2.85462448 | 1E-10 | 3.6533E-09 | 1.986E-09 |
| GO:0005681 | spliceosomal complex | 158 | 0.585577914 | 2.7875836 | 1E-10 | 3.6533E-09 | 1.986E-09 |
| GO:0030133 | transport vesicle | 98 | 0.557982255 | 2.49782931 | 1E-10 | 3.6533E-09 | 1.986E-09 |
| GO:0010008 | endosome membrane | 235 | 0.425320257 | 2.14044081 | 3.0086E-10 | 1.0304E-08 | 5.6015E-09 |
| GO:0031982 | vesicle | 153 | 0.472194295 | 2.24923735 | 7.5312E-10 | 2.4277E-08 | 1.3197E-08 |
| GO:0005758 | mitochondrial intermembrane space | 81 | 0.57124294 | 2.45018989 | 9.7403E-10 | 2.9654E-08 | 1.612E-08 |
| GO:0043202 | lysosomal lumen | 91 | 0.552357711 | 2.44331191 | 1.463E-09 | 4.2195E-08 | 2.2937E-08 |
| GO:0031966 | mitochondrial membrane | 151 | 0.466213189 | 2.21572429 | 2.0583E-09 | 5.6398E-08 | 3.0658E-08 |
| GO:0035578 | azurophil granule lumen | 80 | 0.559822842 | 2.39001835 | 2.4387E-09 | 6.3639E-08 | 3.4594E-08 |
| GO:0000502 | proteasome complex | 63 | 0.601136725 | 2.44820224 | 3.2987E-09 | 8.2168E-08 | 4.4667E-08 |
| GO:0071005 | U2-type precatalytic spliceosome | 50 | 0.637848345 | 2.49953746 | 8.041E-09 | 1.9158E-07 | 1.0415E-07 |
| GO:0005793 | endoplasmic reticulum-Golgi intermediate compartment | 80 | 0.544170755 | 2.32319582 | 1.3807E-08 | 3.1525E-07 | 1.7137E-07 |
| GO:0005801 | cis-Golgi network | 47 | 0.641346361 | 2.46618368 | 1.4403E-08 | 3.1571E-07 | 1.7162E-07 |
| GO:0045211 | postsynaptic membrane | 249 | -0.361484434 | -1.98050655 | 1.7553E-08 | 3.6997E-07 | 2.0112E-07 |
| GO:0009897 | external side of plasma membrane | 262 | -0.343693917 | -1.89661935 | 2.2195E-08 | 4.5048E-07 | 2.4488E-07 |
| GO:0062023 | collagen-containing extracellular matrix | 338 | 0.360982546 | 1.88159593 | 5.6492E-08 | 1.1056E-06 | 6.0102E-07 |
| GO:0005763 | mitochondrial small ribosomal subunit | 29 | 0.708888256 | 2.43795533 | 7.6128E-08 | 1.4386E-06 | 7.82E-07 |
| GO:0005741 | mitochondrial outer membrane | 172 | 0.427023205 | 2.06583392 | 8.0072E-08 | 1.4627E-06 | 7.951E-07 |

**Supplementary table 6: CONSORT crossover checklist, GlucoBAT Study**

| Section/topic | Item No | Description | Page No* |
| --- | --- | --- | --- |
| Title† | 1a | Identification as a randomised crossover trial in the title | 1 |
| Abstract† | 1b | Specify a crossover design and report all information outlined in table 2 | 3 |
| Introduction: | | | |
| Background‡ | 2a | Scientific background and explanation of rationale | 5 |
| Objectives‡ | 2b | Specific objectives or hypotheses | 5 |
| Methods: | | | |
| Trial design† | 3a | Rationale for a crossover design. Description of the design features including allocation ratio, especially the number and duration of periods, duration of washout period, and consideration of carry over effect | 6 |
| Change from protocol‡ | 3b | Important changes to methods after trial commencement (such as eligibility criteria), with reasons | NA |
| Participants‡ | 4a | Eligibility criteria for participants | 6 |
| Settings and location‡ | 4b | Settings and locations where the data were collected | 6 |
| Interventions† | 5 | The interventions with sufficient details to allow replication, including how and when they were actually administered | 6 |
| Outcomes‡ | 6a | Completely defined prespecified primary and secondary outcome measures, including how and when they were assessed | 11 |
| Changes to outcomes‡ | 6b | Any changes to trial outcomes after the trial commenced, with reasons | NA |
| Sample size† | 7a | How sample size was determined, accounting for within participant variability | 11 |
| Interim analyses and stopping guidelines‡ | 7b | When applicable, explanation of any interim analyses and stopping guidelines | NA |
| Randomisation: | | | |
| Sequence generation‡ | 8a | Method used to generate the random allocation sequence | 6 |
| Sequence generation‡ | 8b | Type of randomisation; details of any restriction (such as blocking and block size) | 6 |
| Allocation concealment mechanism‡ | 9 | Mechanism used to implement the random allocation sequence§ (such as sequentially numbered containers), describing any steps taken to conceal the sequence until interventions were assigned | 6 |
| Implementation† | 10 | Who generated the random allocation sequence,§ who enrolled participants, and who assigned participants to the sequence of interventions | 6 |
| Blinding‡ | 11a | If done, who was blinded after assignment to interventions (for example, participants, care providers, those assessing outcomes) and how | 6 |
| Similarity of interventions‡ | 11b | If relevant, description of the similarity of interventions | 6 |
| Statistical methods† | 12a | Statistical methods used to compare groups for primary and secondary outcomes which are appropriate for crossover design (that is, based on within participant comparison) | 11 |
| Additional analyses‡ | 12b | Methods for additional analyses, such as subgroup analyses and adjusted analyses | 11 |
| Results | | | |
| Participant flow (a diagram is strongly recommended)† | 13a | The numbers of participants who were randomly assigned, received intended treatment, and were analysed for the primary outcome, separately for each sequence and period | Diagram is provided in supplementary figure 3 |
| Losses and exclusions† | 13b | No of participants excluded at each stage, with reasons, separately for each sequence and period | NA |
| Recruitment‡ | 14a | Dates defining the periods of recruitment and follow-up | 6 |
| Trial end‡ | 14b | Why the trial ended or was stopped | 12 |
| Baseline data† | 15 | A table showing baseline demographic and clinical characteristics by sequence and period | 12 |
| Numbers analysed† | 16 | Number of participants (denominator) included in each analysis and whether the analysis was by original assigned groups | 12 |
| Outcomes and estimation† | 17a | For each primary and secondary outcome, results including estimated effect size and its precision (such as 95% confidence interval) should be based on within participant comparisons.¶ In addition, results for each intervention in each period are recommended | See suppl. Table |
| Binary outcomes‡ | 17b | For binary outcomes, presentation of both absolute and relative effect sizes is recommended | NA |
| Ancillary analyses‡ | 18 | Results of any other analyses performed, including subgroup analyses and adjusted analyses, distinguishing prespecified from exploratory |  |
| Harms† | 19 | Describe all important harms or untended effects in a way that accounts for the design (for specific guidance, see CONSORT for harms32) | A supplementary table is provided |
| Discussion: | | | |
| Limitations† | 20 | Trial limitations, addressing sources of potential bias, imprecision, and if relevant, multiplicity of analyses. Consider potential carry over effects | 21 |
| Generalisability‡ | 21 | Generalisability (external validity, applicability) of the trial findings | 21 |
| Interpretation‡ | 22 | Interpretation consistent with results, balancing benefits and harms, and considering other relevant evidence | 21 |
| Other information: | | | |
| Registration‡ | 23 | Registration number and name of trial registry | 6 |
| Protocol‡ | 24 | Where the full trial protocol can be accessed, if available |  |
| Funding‡ | 25 | Sources of funding and other support (such as supply of drugs), role of funders | 24 |
