# Supplementary figures and images for: Effect of high-dose glucocorticoid treatment on human brown adipose tissue activity: a randomised, double-blinded, placebo-controlled cross-over trial in healthy men
Source: eBioMedicine. 2023 Sep 4;96:104771. doi: 10.1016/j.ebiom.2023.104771 (PMC10483510; doi:10.1016/j.ebiom.2023.104771)

# DHEAS

$<0.0001$

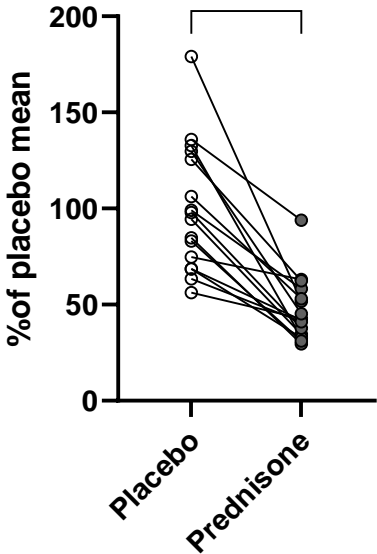

Supplement: Supplementary Fig. S1 [file mmc2.pdf]

# Supplementary Figure 2

**A**

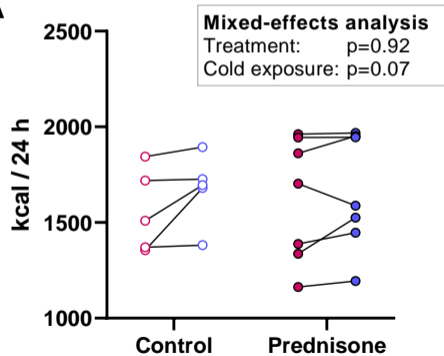

**B**

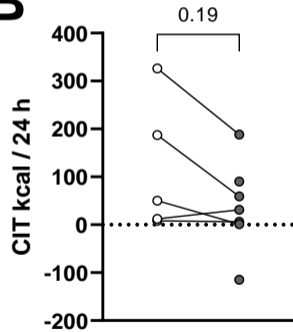

**C**

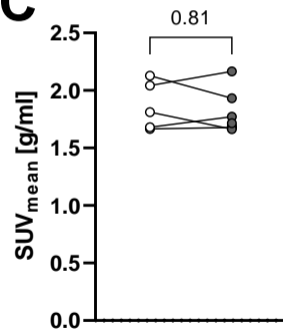

- Control
- Prednisone
- warm
- cold

Supplement: Supplementary Fig. S2 [file mmc3.pdf]

# Supplementary Figure 3

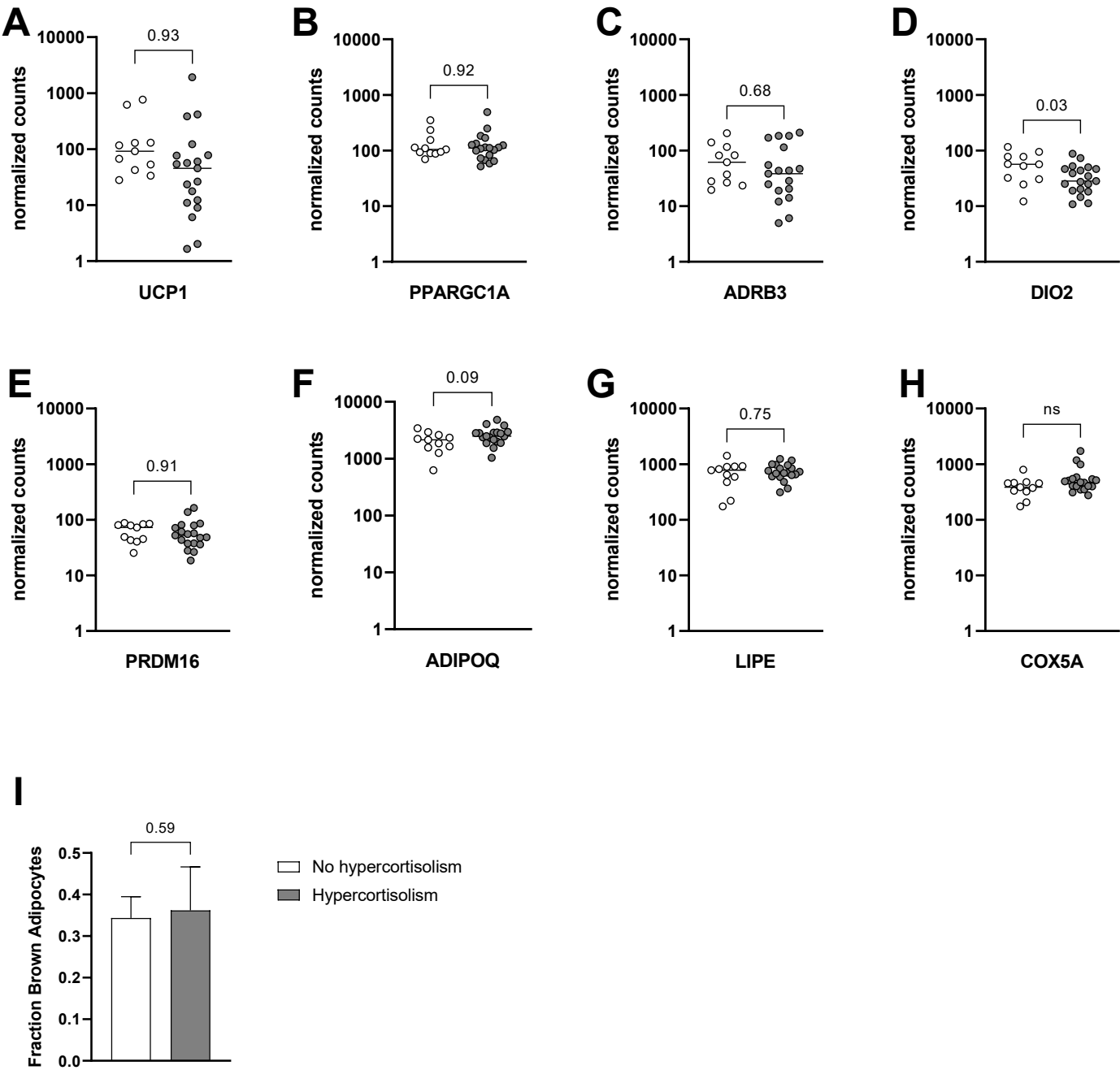

Supplement: Supplementary Fig. S3 [file mmc4.pdf]
